# Success/failure condition influences attribution of control, negative affect, and shame among patients with depression in Singapore

**DOI:** 10.1186/s12888-017-1451-7

**Published:** 2017-08-02

**Authors:** Si-Ning Yeo, Hani Zainal, Catherine S. Tang, Eddie M. Tong, Cyrus S. Ho, Roger C. Ho

**Affiliations:** 1 Duke-NUS Medical School, Neuroscience and Behavioral Disorders Program, 8 College Road, Singapore, 169857 Singapore; 2 Pennsylvania State University, 378 Moore Building, University Park, PA 16801 USA; 3 Department of Psychology, Faculty of Arts and Social Sciences, National University of Singapore, Block AS4 Level 2, 9 Arts Link, Singapore, 117570 Singapore; 4 Department of Psychological Medicine, Yong Loo Lin School of Medicine, National University of Singapore, 1E Kent Ridge Rd, Singapore, 119228 Singapore

**Keywords:** Major depressive disorder, Attribution of control, Shame, Asia

## Abstract

**Background:**

There remains a paucity of research on control attribution and depression within Asian populations. This study examines: (1) Success/Failure condition as a moderator between depression and negative affect or shame, and (2) differences in control attribution between patients with depression and healthy controls in Singapore.

**Methods:**

Seventy one patients with depression and 71 healthy controls went through a digit-span memory task where they were randomized into either the Success or Failure condition. Participants in the Success condition had to memorize and recall 5-digit strings, while participants in the Failure condition did the same for 12-digit strings. They then completed self-report measures of negative affect, shame, and attribution of control. One-way ANCOVA was performed to examine task condition as a moderator of association between mental health status and post-task negative affect or shame. Test of simple effects was carried out on significant interactions. Sign test and Mann-Whitney U test were employed to investigate differences in attribution of control.

**Results:**

Mental health status and Success/Failure condition had significant effects on reported negative affect and shame. Healthy controls reported less post-task negative affect and shame in the Success than in the Failure condition while patients with depression reported similar levels of post-task negative affect and shame in both conditions. However, these differences were not significant in the test of simple effects. In addition, healthy controls felt a stronger sense of personal control in success than in failure and were more likely to blame external factors in failure than in success. Conversely, patients with depression were more inclined to credit external factors in success than in failure and ascribed greater personal control in failure than in success.

**Conclusion:**

The results suggest that successful conditions may not necessitate the reduction of negative affect in Asians with depression, indicating possible cultural variation in affective states as a result of control attribution and the importance of attending to these variations in designing psychological intervention for Asians. Further studies are required to gather more evidence on control attributions in different contexts and study other cognitive mechanisms related to depression in the Asian population.

## Background

Major Depressive Disorder (MDD) is a leading cause of disability and carries the heaviest burden of disease among mental and behavioral disorders worldwide [1]. Individuals with MDD are not only likely to have low psychological adjustment but also impaired functioning in personal relationships [2, 3], work [4] and health [5, 6]. The incremental economic burden [7] and high mortality risk [8] makes MDD an extremely pressing issue. Taking into account that socio-cultural factors impact the phenomenology and treatment of MDD, the literature is in dire need of a more comprehensive Asian perspective to refine the understanding and treatment of MDD in the Asian context. The present study attempts to do so by examining depression, negative affect, and success/failure attribution within an Asian sample.

### Depression, negative affect and shame

Emotion-based theories of psychopathology posit that, compared to the normal population, people with depression experience more negative affect [9, 10]. Shame, in particular, is a negative affect characteristic of depression [11, 12]. It is experienced when one fails to meet social or moral standards [13] and includes feelings of powerlessness, self-consciousness, low self-esteem, and low self-worth [14, 15].

### Depression, shame and success/failure attribution

Cognitive mechanisms play an important role in the experience of emotion [16]. It is thus valuable to explicate the cognitive mechanisms that predict negative affect in depression. One type of cognitive mechanism is cognitive attribution of control. Success/Failure attribution of control along the internal/external continuum influences the amount of shame experienced by the individuals [17, 18] and is linked to depression. According to the cognitive theory of depression [11], individuals with depression are inclined toward assuming personal responsibility for undesirable events, thus giving rise to feelings of shame. They may also view positive conditions as uncontrollable and consequent of external factors or pure luck [19]. On the other hand, individuals without depression tend to engage in self-serving attributional bias, ascribing positive conditions to themselves and negative conditions to transient internal psychological states or external agents or causes [20].

### Purposes of the study and hypotheses

Despite the wealth of research examining control attribution in relation to depression in Western samples, there are no known similar studies in Asian populations. However, cross-cultural researchers have put forth the idea that the attributional style of sharing responsibility for success and taking responsibility for failure as being the most adaptive way to maintain self-esteem in the community since the self is perceived as part of a network of social relationships in the Asian culture [21, 22]. Hence, we can expect Asians to attribute success to both internal and external factors and feel more positive affect since sharing credit for success is congruent with the culture of preserving social relationships. On the other hand, we postulate Asians with depression to experience significantly more negative affect in an unfavorable situation as they would attribute negative outcomes to internal factors and thus bring their sense of self-consciousness and low self- esteem into focus.

As yet, no published study has tested this explicitly through an experimental study simulating success and failure conditions in Asian clinical and healthy samples. Therefore, we aimed to investigate the difference in reported negative emotion and attributions of control between Asians with depression and healthy participants after completing a task in which they either succeeded or failed in. We expected the Success/Failure condition to moderate the association between depression and negative affect, with an emphasis in shame.

Our specific hypotheses were:

- Hypothesis 1: Patients with depression would report more negative affect and shame than healthy controls at post-task in Success and Failure conditions.
- Hypothesis 2: Patients with depression in Failure condition would report more post-task negative affect and shame than patients in Success condition.
- Hypothesis 3: Patients with depression would attribute their failure to internal factors and success to external factors. Conversely, we expected healthy controls to attribute external factors for failure and internal factors for success.

## Methods

We conducted a 2 (Mental Health Status: patients with depression vs healthy controls) × 2 (Condition: Success vs Failure) between subjects design.

### Participants

A total of 145 participants (72 patients with depression and 73 healthy controls) were recruited for the study. The final analysis included data from 71 patients and 71 healthy controls. 3 participants (1 patient and 2 healthy controls) were excluded as their data points were significant outliers, thus violating the assumptions required for our analysis.^1^

Participants who were between the ages of 30–60 and had a minimum of six years of education were considered for inclusion. Additionally, healthy controls were recruited if they did not have any history of psychiatric disorder. These controls were recruited from the community by word-of-mouth. All patients were diagnosed using the Structured Clinical Interview for the Diagnostic and Statistical Manual of Mental Disorders (DSM) by psychiatrists from a local hospital. They had a diagnosis of MDD at the time of study with no other comorbid mental conditions. Bipolar disorder, schizophrenia, substance abuse were also assessed and ruled out. These patients were either receiving inpatient or outpatient treatment for depression at the hospital. They were referred to the study by their treating psychiatrists. There was no incentive given for participation in the study. The research protocol took 30 min to complete. Ethics approval was obtained from both the hospital and university ethics review boards.

### Measures

#### Demographics

Demographics such as age, gender, ethnicity, marital status and housing type were collected from all participants.

#### Negative affect and shame

The 10 items measuring negative affect in the Positive and Negative Affect Schedule (PANAS) [23] were used to measure negative affect, including shame. It is a self-report measure to indicate the extent to which the respondent is feeling each emotion. Each item is rated on a 5-point scale ranging from 1-very slightly or not at all to 5- extremely. The internal consistency of the PANAS was high (α = .93).

#### Level of depression

The Depression, Anxiety and Stress Scales (DASS) [24] were used to determine the level of depression in the participants. Only the scales for depression and anxiety were used to give a 14-item subscale as stress was not a critical variable of interest. Items are rated on a 4-point scale using a time-frame of ‘over the past week’. The internal consistency of the scale was excellent (α = .95).

#### Attribution of control

To understand the attribution of control participants had for the condition of the task, a scale that included items related to sense of control was used. The items were adapted and revised from the questionnaires used by Ellsworth and Scherer [25]. Items measured control in the domains of “self” (e.g. I influence how well I do on this task), “other” (e.g. Other people (e.g. the research assistant) influence how well I do on this task) or “situation” (e.g. My surroundings influence how well I do on this task).

There was a total of 6 items in this scale. The items were rated on a scale from 1-not at all to 9-very much. Internal consistency of items for each domain was high with Cronbach’s alphas above .80.

## Procedures

All participants provided written informed consent before taking part in the study. They were subsequently randomized into either the Success or Failure condition. Next, the participants filled out the PANAS before the research assistant explained the instructions of the memory task. The memory task was administered via a computer by the research assistant. A digit-string was flashed on the screen for 3 s and then participants had 20 s to recall the digit-string on a piece of paper. There was a total of 3 trials in the memory task. In the Success condition, participants were given a 5 digit-string to memorize in each trial. In the Failure condition, participants were given a 12-digit string to memorize in each trial. The research assistant observed the participant as he/she completed the task. After the memory task, all participants in the Success condition were told that they had passed, while all participants in Failure condition were told that they had failed. This was regardless of their actual performance. The participants then completed the PANAS scale for the second time, followed by the attribution of control scale and the DASS. Upon completion, the participants were debriefed on the purpose of the study. This was a once off participation with no follow-up activities.

### Statistical analysis

Data analysis was performed using IBM Statistical Package for the Social Sciences (SPSS) 23. The internal consistency of the measures and descriptive statistics were generated first. Chi-Square test and independent T-test was then conducted to compare differences in demographics between patients and healthy controls. Mann-Whitney U test was used to compare pre-task PANAS scores between groups and conditions. Sign test and Mann-Whitney U test was employed to examine differences in attribution of control. One-way ANCOVA was later performed to examine task condition as a moderator of association between mental health status and post-task negative affect or shame, controlling for pre-task negative affect/shame scores. Test of simple effects was carried out on significant interactions.

## Results

### Sample characteristics

The patients with depression and healthy controls and were equivalent on several background demographic characteristics (Table 1). The two groups did not differ significantly in terms of age, gender, ethnicity and marital status. However, there were significant differences in the level of education and housing type. As expected, patients with depression had significantly more depressive and anxiety symptoms than healthy controls.

**Table 1:** Summary of key demographic information of participants

	Patients with depression (*n* = 71)	Healthy Controls (*n* = 71)	Statistics	*p*-value
	N (%) or Mean (SD)	N (%) or Mean (SD)		
Condition				
Success	35 (49.3%)	34 (47.9%)	χ^2^(1) = .03	.87
Failure	36 (50.7%)	37 (52.1%)		
Age	41.71 (12.9)	38.82 (11.8)	*t*(138) = 1.39	.17
Gender				
Female	40 (56.3%)	43 (60.6%)	χ^2^(1) = .26	.61
Male	31 (43.7%)	28 (39.4%)		
Ethnicity				
Chinese	52 (73.2%)	51 (71.8%)	χ^2^(2) = 8.03	.09
Malay	7 (9.9%)	14 (19.7%)		
Indian, Caucasian and others	12 (16.9%)	6 (8.5%)		
Education				
High School or below	31 (43.7%)	12 (16.9%)	χ^2^(1) = 22.74	.000^***^
Pre-University or above	33 (46.5%)	59 (83.1%)		
Housing Type				
1–2 to 3–room flat	22 (31.0%)	7 (9.9%)	χ^2^(1) = 10.55	.014^**^
4–5 room flat or private housing	49 (69.0%)	64 (90.1%)		
Living arrangement				
Living alone	8 (12.7%)	3 (4.2%)	χ^2^(1) = 2.46	.12
Not living alone	63 (88.7%)	68 (95.8%)		
Marital status				
Married	37 (52.1%)	42 (59.1%)	χ^2^(2) = .98	.61
Single	29 (40.8%)	26 (36.6%)		
Divorced	5 (7.0%)	3 (4.2%)		
DASS				
DASS Anxiety	7.72 (5.37)	2.38 (2.16)	*t*(140) = 7.77	.000^***^
DASS Depression	9.86 (6.22)	2.21 (2.88)	*t*(140) = 9.40	.000^***^

^**^ *p* < .05 ^***^ *p* < .001

### Manipulation checks

Most participants in the Success condition recalled the digit strings accurately in all three trials (92.9%) while none of the participants (0%) in the Failure condition managed to do so.

Before the memory task, there was no significant difference in reported negative affect between participants in Success and Failure conditions in both the healthy controls (Success: Mdn = 10, Failure: Mdn = 11, *U* = 533.5, *p* = .228, *r* = − .14) and patients with depression (Success: Mdn = 22, Failure: 17.5, *U* = 472.5, *p* = .07, *r =* − .21). Similarly, there was no significant difference in pre-task reported shame ratings between participants in both conditions in both healthy controls (Success: Mdn = 1, Failure: Mdn: 1, *U* = 580.0, *p* = .275, *r* = − .13) and patients with depression (Success: Mdn = 2, Failure: 1, *U* = 536.5, *p* = .24, *r =* − .14).

### Task condition as a moderator between mental health status and post-task negative affect

There was a significant main effect of mental health status on reported post-task negative affect, *F* (1137) = 9.45, *p = .*003, *d* = 0.11, and a significant main effect of task condition on reported post-task negative affect, *F* (1137) = 1.13, *p =* .008, *d* = 0.18, after controlling for pre-task negative affect ratings. Further, there was a significant interaction between mental health status and task condition on reported post-task negative affect, *F* (1137) = .331, *p = .*002, *d* = 0.10.

**Fig. 1:**
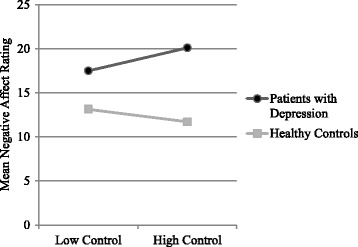
Mean post-task negative affect rating by mental health status and task condition

Patients with depression in the Success condition (*M* = 20.11, *SD* = 10.10) reported similar levels of negative affect as patients in the Failure condition (*M* = 17.50, *SD* = 7.77), *t* (69) = 1.22, *p* = .069. Conversely, healthy controls in the Success condition (*M* = 11.18, *SD* = 2.37) reported less negative affect than healthy controls in the Failure condition (*M* = 13.13, *SD* = 4.86), *t* (69) = −2.13, *p* = .037, *d* = 0.51. However, the test of simple main effects showed that the difference in negative affect reports were not significant (Success: *F*(1137) = .015, *p* = .904, Failure: *F*(1137) = .840, *p* = .361) (Fig. 1).

### Task condition as a moderator between mental health status and post-task shame

There was a significant main effect of mental health status on reported post-task shame *F* (1137) = .286, *p = .*002, *d* = 0.09 and a significant main effect of task outcome on post-task shame after controlling for pre-task shame ratings, *F* (1137) = 1.44, *p =* .01, *d* = 0.20. The interaction effect between mental health status and task outcome on post-task shame was also significant, *F* (1137) = 1.394, *p = .*01, *d* = 0.20.

**Fig. 2:**
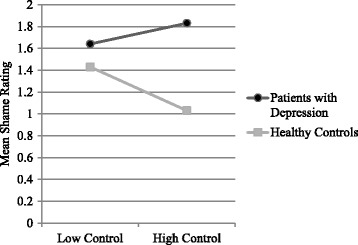
Mean post-task shame rating by mental health status and task controllability

Healthy controls in the Success condition (*M* = 1.03, *SD* = .17) reported less shame than those in the Failure condition (*M* = 1.43, *SD* = .93), *t* (69) = −2.49, *p* < .001, *d* = 0.60. However, there was no significant difference in shame ratings among patients with depression across conditions (Success: *M* = 1.83, *SD* = 1.24, Failure: *M* = 1.64, *SD* = 1.17, *t* (69) = .66, *p* = .619). The test of simple main effects showed that these differences in shame ratings were not significant (Success: *F* (1137) = 1.31, *p* = .253, Failure: *F*(1137) = 1.32, *p* = .673) (Fig. 2). Therefore, Hypothesis 1 and Hypothesis 2 were not supported.

### Differences in control attribution

There was no significant difference in ratings between personal and external attribution of control for patients in the Failure condition, Z < .00, *p* > .05, as well as healthy controls in the Success condition, Z = −.50, *p* = .617. Similar findings were made in the Success condition for both the patients, Z = −1.39, *p* = .164, and healthy controls, Z = .00, *p* > .05.

However, patients with depression in the Failure condition (Mdn = 7) felt more personally responsible for the condition than those in the Success condition (Mdn = 5), *U* = 415.0, *p* = .013, *r* = −.57. There was no significant difference in external attribution of control between Success (Mdn = 6) and Failure conditions (Mdn = 3) for the patients with depression, *U* = 475.0, *p* = .066, *r* = −.22.

On the other hand, healthy controls in the Success condition (Mdn = 8.5) reported a higher sense of personal control over their performance in the task as compared to healthy controls in the Failure condition (Mdn = 6.5), *U* = 213.5, *p* = .000, *r* = −.57. However, there was no significant difference in attribution of external control between healthy controls in the Success (Mdn = 5) and Failure (Mdn = 6.5) conditions, *U* = 553.0, *p* = .377, *r* = −.09.

In the Success condition, healthy controls (Mdn = 8.5) felt that they had more personal control of their scores than patients with depression did (Mdn = 7), *U* = 328.5, *p* = .001, *r* = −.39. In the Failure condition, healthy controls (Mdn = 6.5) felt more strongly that external factors were responsible for their failure as compared to patients with depression (Mdn = 3), *U* = 443.5, *p* = .012, *r* = −.30. Hypothesis 3 was thus supported.

## Discussion

Results on the attribution of control in the present study supported the idea that Asians have a proclivity to share responsibility across success and failure conditions. Yet, there were also salient differences in the way patients with depression and healthy controls accredited their success and failure to, and this was largely congruent with extant literature on self-serving attribution bias. Patients with depression blamed themselves for their poor performance in the failure condition but were resistant to the idea that they scored well in the success condition due to their own abilities. This is characteristic of the depressive attributional style suggested by attributional models of depression [26, 27]. Attributions to internal, stable, and global causes for negative events have a reliable and significant association with depression while attributions to external, unstable, and specific causes for positive events are associated with depression [28]. Further, placing patients with depression in a situation of success did not decrease their negative feelings or experience of shame but maintained it. Markus and Kitayama [29] have suggested that individuals in a collectivist culture, which is characteristic of an Asian society like Singapore, are more likely to engage in self-criticism than in self-promotion. We postulate that in the Success condition, by attributing their success to external instead of internal factors, self-criticism may have exacerbated their feelings of inadequacy and worthlessness and thus resulted in the maintenance of shame and negative affect. Taken together, the attributional style found in the Asian MDD patients may be exceptionally dysfunctional and debilitating because even positive events like being successful in a memory task, which should otherwise lighten their mood, elicit the same level of negative affect as being in a failure condition. People with depression already experience higher levels of negative affect and self-criticism, and it does not help that their attributional reactions to both success and failure conditions perpetuate their negative feelings and shame. This could potentially permit and sustain the integration of bad but not good outcomes in the structure of beliefs about themselves [28] and maintain their depression. However, as task condition did not significantly influence the negative affect and shame scores in both groups, we interpret these results with caution and recommend further exploration in future studies to support our current findings.

### Strengths and limitations

To the authors’ knowledge, no published study has compared Success/Failure attributions of control in clinical and non-clinical populations in the Asian context. Furthermore, the present study recruited participants from the clinical population for the depressive sample and extended recruitment of healthy controls beyond the college population for greater external validity.

Despite our efforts, there were still some limitations in our study design. For one, the attributional explanation of our participants may not be cross-situationally consistent. The presence of a research assistant in the room may also have influenced their self-reports of negative affect, shame, and attribution of control. It is important for future studies to explore the same constructs in other situations and at different time-points to further validate the present findings.

Secondly, the small effect sizes and lack of significant results in the test of simple effect suggest that a third variable might have contributed to the obtained results. Some possible examples include cognitive variables like negative beliefs about self and depressive thought frequency, anxiety, and sense of self-efficacy. While we did not test these variables in our study, the premise and findings set the stage for future investigations to build a more robust paradigm for understanding negative affect experience across situations in the clinical population.

Lastly, including a Western comparison group would also have been helpful in providing a complete knowledge of cultural differences in attributional style and emotional experience. This should be considered in future studies exploring similar constructs.

### Clinical implications

Reattribution training has been employed to help clients work on reattributing causes of failure to lack of effort, utilizing their sense of personal control and be motivated to increase their self-efficacy [30]. Similarly, self-efficacy of patients with depression can be strengthened with reattribution training for positive conditions. Enabling Asian patients with depression to reattribute positive events to internal factors could help alleviate symptoms and decrease negative affect. However, additional research will be required and clinicians and researchers will have to tread forward mindfully to study the efficacy of interventions targeting attributional styles in Asian patients for culturally sensitive management of depressive disorders.

## Conclusion

Our results suggest that the way Asians attribute internal and external control to success/failure conditions, while similar to the literature on Western counterparts, may have pernicious effects on affective states and depressive symptoms as a successful condition did not alter the reported levels of negative affect in participants with depression. Further research that identifies the underlying mechanisms and factors that influence the relationship between attributional style and affective state in Asian samples can help ascertain that. Additionally, future research should examine attributional styles across different contexts in Asian populations to gain a clearer understanding of the cognitive mechanisms of depression unique to this population. This can then aid in tailoring psychotherapy specific to the needs of Asian clients.

## Abbreviations

DASS: Depression, Anxiety and Stress Scales
DSM: Diagnostic and Statistical Manual of Mental Disorders
IBM SPSS: IBM Statistical Package for the Social Sciences
MDD: Major Depressive Disorder
NHG DSRB: National Healthcare Group Domain Specific Review Board
NUS IRB: National University of Singapore Institutional Review Board
PANAS: Positive and Negative Affect Schedule

## Footnotes

1 Excluding the outliers from the analysis did not change the results.
